# Targeted Biocatalyst Design for Asymmetric Citalopram Conversion in a Membrane Reactor

**DOI:** 10.3390/pharmaceutics17111497

**Published:** 2025-11-19

**Authors:** Oliwia Degórska, Natalia Zasada, Weronika Badzińska, Qiang Fu, Teofil Jesionowski, Jakub Zdarta

**Affiliations:** 1 Institute of Chemical Technology and Engineering, Faculty of Chemical Technology, Poznań University of Technology, Berdychowo 4, 60965 Poznań, Poland; nataliazasada16@gmail.com (N.Z.); weronika.badzinska@doctorate.put.poznan.pl (W.B.); teofil.jesionowski@put.poznan.pl (T.J.); 2 School of Civil and Environmental Engineering, University of Technology Sydney, Ultimo, NSW 2007, Australia; qiang.fu@uts.edu.au

**Keywords:** biocatalysis, enzyme immobilization, biocatalytic API resolution

## Abstract

**Objective:** This study aimed to develop a stable and active biocatalytic system for enzyme immobilization, utilizing an electrospun support doped with a metal–organic framework (MOF) and supplemented with an ionic liquid as a lipase stabilizer and activity enhancer. **Methodology:** The system was applied for an efficient and enantioselective resolution of racemic citalopram. Key parameters, including MOF concentration, electrospinning and immobilization conditions, ionic liquid selection, and reaction time, were optimized to enhance biocatalyst performance. **Results:** The optimal immobilization time was determined to be 24 h, achieving 52% immobilization efficiency and 100% activity recovery. The resulting biocatalytic system HIGH PVC-MOF-lip-CA exhibited superior storage stability, retaining 80% of its initial activity, a 75% improvement over the free enzyme. In the resolution of citalopram, the system achieved 96% conversion of S-citalopram within 24 h, with the enantiomeric excess of 93% in favor of the S-ester over the R-ester. These findings demonstrate the system’s potential for efficient and stereoselective biocatalytic applications.

## 1. Introduction

The increasing demand for sustainable and efficient chemical processes has driven the development of innovative solutions to enhance productivity while minimizing environmental impact. Among these advancements, hybrid materials, which synergistically integrate organic and inorganic components, have emerged as a promising approach due to their unique and tunable properties [1]. By combining the advantages of different material classes, these systems offer tailored functionalities that can address key challenges in catalysis, separation, and biocatalysis. Metal–organic frameworks (MOFs) are characterized as porous structures made of metal ions or clusters connected by organic ligands. Due to their unprecedented design flexibility at the molecular level, it is possible to precisely adjust their properties by modifying both the metallic and organic components. As a result, the materials obtained have controlled porosity, pore size and shape, hydrophilicity, required functional groups, and other specific chemical properties [2,3]. In addition, their optical, catalytic, as well as magnetic properties are also attracting a great deal of attention from researchers [4,5]. For this reason, these unique properties offer benefits in the fields of catalysis, gas separation, environmental purification, and analytical detection [6]. Nonetheless, the opportunity to utilize MOFs in biocatalysis is particularly appealing. By immobilizing enzymes in their structures, it is not only possible to improve the stability of catalytic proteins, but also to control their activity through a suitably designed and controlled microenvironment [7].

Additional possibilities for optimizing catalytic properties and increasing the efficiency and productivity of biocatalytic processes are offered by combining MOFs with other components, such as ionic liquids (ILs). Referred to as ‘designer solvents’, ionic liquids owe their name to their configurable chemical nature, which directly affects the enhanced stability and performance of immobilized enzymes [8,9,10]. They are distinguished by their negligible vapor pressure, high stability in both chemical and thermal conditions, as well as high ionic conductivity. Because of these characteristics, ionic liquids used in enzyme catalysis exhibit the ability to stabilize the quaternary structure of enzymes, thereby contributing to their durability and resistance to extreme pH and temperature conditions. Consequently, this makes it possible to limit denaturation processes and allows reactions to proceed under conditions that would be destructive to free enzymes [11]. The application of the components aligns perfectly with the enzymatic synthesis of active pharmaceutical ingredients. The use of enzymes for such reactions fits within the principles of green chemistry and sustainable development goals, offering mild reaction conditions and low environmental toxicity. An additional advantage is the high selectivity of these reactions, which is crucial for obtaining specific enantiomers of active compounds, as exemplified by citalopram. To date, most biocatalytic processes rely predominantly on the use of free enzymes, which, despite their effectiveness, suffer from several limitations, including low stability, lack of reusability, and poor resistance to changing environmental conditions.

To overcome limitations of free enzymes, immobilization is the most frequently used solution. There have been studies and examples in the scientific literature on the efficient use of such immobilized systems in the separation of racemic mixtures as well as the synthesis of APIs. In one of the presented research projects, a *Candida antarctica* lipase immobilized in a MOF matrix was utilized, enabling efficient separation of carprofen enantiomers, achieving high yields and good selectivity. In addition, the produced system achieved better pH and temperature tolerance than the free enzyme [12]. In the context of naproxen, it was shown that the addition of ionic liquid as a reaction medium significantly improved the activity and enantioselectivity of lipase in the separation of (R,S)-naproxen, enabling efficient and stable enzymatic separation of the enantiomers [13,14]. In the enantioselective esterification of ibuprofen, *Candida antarctica* B lipase demonstrated remarkable efficiency when paired with the ionic liquid [BMIM][BF_4_], achieving high reaction yields and enhanced selectivity compared to conventional organic solvent systems [15]. Such studies underscore the potential of enzyme-mediated biocatalysis in the pharmaceutical industry, offering a greener and more sustainable alternative to traditional chemical processes.

In this study, we developed a novel MOF-dopped electrospun nanofiber as a support for the immobilization of lipase *Candida* sp., with ionic liquids incorporated as activity enhancer factors. The resulting biocatalytic system was designed for the enantioselective resolution of racemic citalopram, a key pharmaceutical intermediate. By synergistically combining MOFs and ionic liquids, this approach aligns with green chemistry principles, offering a sustainable alternative for enzymatic catalysis in pharmaceutical synthesis, an area of growing research interest.

## 2. Materials and Methods

### 2.1. Reagents and Chemicals

Poly(vinyl chloride) (PVC) with low (MW = 40 kDa) and high (MW = 125 kDa) molecular weights, zirconium(IV) chloride (ZrCl_4_), 2-aminoterephthalic acid (NH_2_-BDC), acetic acid, N,N-dimethylformamide (DMF), dichloromethane (DCM), lipase from *Candida* sp., choline acetate, choline chloride, paranitrophenyl palmitate (pNPP), vinyl acetate, acetonitrile, and used buffer solutions were purchased from Sigma Aldrich (Darmstadt, Germany) and used as received. Racemic mixture of citalopram was purchased from Angene (Nanjing, China).

The MOF was prepared via a solvothermal reaction by dissolving zirconium (IV) chloride (ZrCl_4_) and 2-aminoterephthalic acid (NH_2_-BDC) (500 mg) in anhydrous N,N-dimethylformamide (DMF) (30 mL) at a 1:1 molar ratio, with acetic acid (2 mL) added as a crystallization modulator. The mixture was sonicated for homogeneity, transferred to a Teflon-lined autoclave, and heated at 120 °C for 24 h. After cooling, the resulting yellow crystalline product was isolated by centrifugation, washed sequentially with DMF and ethanol to remove unreacted precursors and residual solvents, and finally vacuum-dried at 80 °C to yield activated UiO-66-NH_2_ (with a zirconium-based metal–organic framework with amino functional groups, enabling further interactions).

### 2.2. Analytical Methods for the Morphology Evaluation

For the confocal laser scanning microscopy, two modes were used in this study: material mode, which recorded light reflection from the surface, and fluorescence mode, which recorded light emission. A Zeiss LSM710 confocal microscope (ZEISS, Oberkochen, Germany) was used for the analysis, built in an upright configuration on an Axio Imager Z2m stand (ZEISS, Oberkochen, Germany). The objective lens used was an EC Epiplan-Neofluar 20x/0.50 HD DIC (ZEISS, Oberkochen, Germany) with a working area of 0.424 × 0.424 mm. In material mode, laser light with a wavelength of 458 nm was used as illumination, and 488 nm in the fluorescence mode. Luminance observation was set within the wavelength range of 510–797 nm.

Thermogravimetry was used to visualize the change in sample mass caused by temperature changes. The results of this analysis are presented in the form of a thermogram. Measurements were conducted in nitrogen, air, argon, oxygen, or vacuum atmospheres, and the sample mass typically decreased with increasing temperature. The thermal stability of the materials was assessed using a Jupiter STA 449 device from Netzsch GmbH (Selb, Germany).

The electrokinetic properties of the fabricated membranes were examined over a pH range of 3–11 using zeta potential measurements (Anton Parr SurPASS 3 zeta potential analyzer, Warsaw, Poland). The measurements were evaluated at a 100 μm gap height using a 10^−3^ M KCl solution, and an average of 4 readings was taken and reported at each pH value. Then, 0.05 M of HCl and NaOH solutions was used to adjust the pH of the KCl solution.

To determine the activity of the obtained biocatalysts and to perform Bradford analysis, the solutions were analyzed using UV-Vis spectroscopy. The study utilized the electromagnetic spectrum between 200 and 780 nm. Measurements for the Bradford analysis were performed at a wavelength of 595 nm, while measurements for the biocatalyst activity were carried out at a wavelength of 410 nm on a Jasco V-750 spectrophotometer (Jasco, Tokyo, Japan).

To determine the progress of rac-citalopram resolution, HPLC analysis was conducted using a high-performance liquid chromatograph 1290 Infinity II (Agilent Technologies, Santa Clara, CA, USA) equipped with a diode array detector (DAD). Separation of the enantiomers of the substrate and enzymatic reaction products was performed using an InfinityLab Poroshell 120 Chiral-V column, 2.7 μm, 4.6 × 150 mm (Agilent Technologies, Santa Clara, CA, USA), maintained at a temperature of 15 °C. The mobile phase consisted of a 2 mM ammonium acetate solution with 0.01% acetic acid in methanol. The flow rate of the mobile phase was set at 0.4 mL/min. Detection of analytes was carried out at a wavelength of 230 nm.

### 2.3. Fabrication of the Electrospun Nanofibers

Two solutions of low-molecular-weight poly(vinyl chloride) (LOW PVC) and high-molecular-weight poly(vinyl chloride) (HIGH PVC) were prepared at a concentration of 15% (*w*/*w*) in a mixture of DCM:DMF (15 mL) (1:1 *v*/*v*). The solutions were placed in glass vials for 24 h on a magnetic stirrer (IKA RH basic). Then, to one solution of each type of PVC, MOF (UiO-66-NH_2_) grounded in a mortar was added, so that its percentage in the solution was 3% (*w*/*w*) (relative to the dry mass of the polymer). The solutions with the addition of MOF were again placed on a magnetic stirrer until the MOF was completely dispersed in the polymer mixture. Then, each of the solutions was transferred to 5 mL syringes, which were placed in a syringe pump, which was part of the Spinbox electrospinning kit (Bioinica, Valencia, Spain), and the electrospinning process was started. Four types of electrospun mats were produced, which were used as an organic carrier in the immobilization process fabricated from (i) low-molecular-weight poly(vinyl chloride) solution (LOW PVC), (ii) low-molecular-weight poly(vinyl chloride) solution with the addition of UiO-66-NH_2_ (LOW PVC MOF), (iii) high-molecular-weight poly(vinyl chloride) solution (HIGH PVC), and (iv) high-molecular-weight poly(vinyl chloride) solution with the addition of MOF UiO-66-NH_2_ (HIGH PVC MOF).

The flow rates of the solutions during the electrospinning processes were controlled by the syringe pump settings and were 0.25 mL/h for solutions with the addition of MOF UiO-66-NH_2_ and 0.5 mL/h for pure PVC solutions, while the current voltage during electrospinning was 12 kV for solutions with the addition of MOF UiO-66-NH_2_ and 13 kV for pure PVC solutions. The electrospinning duration of individual mats varied from 15 to 30 min, depending also on the atmospheric conditions affecting the quality of nanofiber formation, since humidity influences the conductivity of the used solution. The variable values influencing the electrospinning process of mats were polymer flow rate controlled using a syringe pump, electrical potential changed using the Spinbox device, and electrospinning time.

### 2.4. Evaluation of MOF Incorporation in the Nanofiber Structure

To test the stability of MOF incorporation in the nanofiber structure, fabricated mats were soaked in a buffer solution, followed by submission for thermogravimetric analysis to estimate the changes in the material composition. A fragment of each mat weighing 10 mg was placed in a 1.5 mL Eppendorf tube, then each sample was flooded with 1 mL of an aqueous solution of phosphate buffer at pH 7. The samples were left on an IKA KS 260 basic shaker for 24 h and then dried in the open air. For comparison, a fragment of each mat weighing 10 mg was also cut out and analyzed without previously immersing it in the buffer. The samples were tested on a Jupiter STA 449 device from Netzsch GmbH (Selb, Germany).

### 2.5. Immobilization Procedure

Lipase from *Candida* sp. at a concentration of 5 mg/mL was placed in glass vials, mixed with an aqueous solution of phosphate buffer at pH 7 in a ratio of 50:50 (2.5 mL:2.5 mL), and then immobilized on carriers—4 types of electrospun mats both with the addition of MOF (UiO-66-NH_2_) and without. The mats used had dimensions of 0.5 × 0.5 cm (the mass of this piece was 2.3 mg ± 0.3 mg). To investigate the effect of ionic liquids on the activity of the obtained biocatalytic systems, 5% aqueous solutions of choline chloride and choline acetate were prepared. They were then added separately to systems containing mat and enzyme in the amount of 25 µL. The following systems were finally obtained: PVC-lip, PVC-lip-CC (addition of choline chloride), and PVC-lip-CA (addition of choline acetate).

To examine the effect of immobilization conditions, the immobilization was performed for 1 h and 24 h. Comparison of these two time points allows us to determine whether extending the immobilization time increases the degree of immobilization and enzyme stability, or whether the process reaches a plateau after a shorter incubation time. Throughout the entire process, the samples were placed on a shaking platform (IKA RH 260 basic), which ensured their continuous mixing at 150 rpm. After a set time, the mats were separated from the reaction mixture and tested for activity, reusability, stability, and immobilization efficiency.

### 2.6. Immobilization Efficiency

In order to determine the immobilization efficiency, Bradford analysis was performed. For this purpose, polymer mats were isolated from the solutions after the immobilization, and the solutions were analyzed. Then, 0.5 mL of each of the 12 solutions was taken and placed in plastic spectrophotometric cuvettes with a capacity of 1.5 mL, and then 0.5 mL of Bradford reagent was added to them. The blank sample was 0.5 mL of phosphate buffer solution at pH 7 and 0.5 mL of Bradford reagent. Analysis was also performed for the enzyme solution, which constituted sample 0 and allowed for the calculation of the percentage efficiency. All solutions were left for 15 min, and then their absorbance was measured on a Jasco V-750 spectrophotometer at a wavelength of 595 nm. The obtained absorbance results, defining the initial amount of enzyme in the solution with a known volume and mass of the carrier, allowed the calculation of the amount of enzyme immobilized in 1 g of carrier, while obtaining the percentage efficiency.

### 2.7. Evaluation of Fabricated Biocatalysts’ Activity

To estimate the activity and stability of fabricated biocatalysts and their optimal conditions for the highest activity, each system was applied for several evaluations. Mats after immobilization were transferred to glass vials with the addition of 1 mL of a 15 mM paranitrophenyl palmitate (pNPP) solution in isopropanol and 2 mL of an aqueous solution of phosphate buffer at a certain pH for the evaluation of the amount of formed product (para-nitrophenol). The reactions took place on a rotational shaker (200 rpm) for 5 min at an estimated temperature, then the reaction was stopped by adding 1 mL of 0.5 M Na_2_CO_3_ solution. After the reaction, all of the obtained solutions were centrifuged in an Eppendorf Centrifuge 5810 R and filtered to separate the precipitate of the formed palmitic acid. After such preparation, its absorbance was measured on a Jasco V-750 spectrophotometer at a wavelength of 410 nm and the concentration of the product was determined based on the calibration curves of para-nitrophenol.

Lipase activity recovery was calculated according to Equation (1):

(1) $$Activity\;recovery\;(\%)=\frac{A_{t}}{A_{i}}\cdot 100\%$$

*A_i_*: the initial activity of lipase added to the immobilization medium,

*A_t_*: the activity of the immobilized lipase.

All the measurements were repeated three times, and the results are presented as a mean value ± standard deviation.

### 2.8. Influence of Process Parameters on Activity of Produced Systems

To determine the pH influence, obtained biocatalysts were tested in different buffer solutions according to the method discussed in Section 2.7. These buffers were phosphate buffer with pH 7 and 8, acetate buffer with pH 5 and 6, and TRIS buffer with pH 9, which were prepared before starting the tests. At each of the above pH values, a reaction was carried out for each type of immobilized material with and without choline acetate (a total of 8 samples) under 40 °C.

To test the influence of temperature on the activity of produced systems, biocatalysts were tested as previously mentioned. The prepared samples were placed in an Eppendorf ThermoMixer C incubator (Warsaw, Poland), which provided the appropriate temperature for the process (20 °C, 30 °C, 40 °C, 50 °C, and 60 °C) at pH 7.

### 2.9. Storage Stability

To carry out the stability test, an electrospun mat was cut into 1 × 1 cm pieces and lipase was immobilized with the addition of choline acetate. The immobilization was carried out for 24 h. Then, each mat was transferred to a separate vial with 5 mL of phosphate buffer at pH 7 and stored at 4 °C for 30 days. Every 3 days (including day 0, the day of receiving the immobilized systems), mat activity tests were carried out by carrying out the pNPP hydrolysis reaction (as described in Section 2.7). The activity of the native enzyme at time 0 was taken as 100% of relative activity, becoming a base for other calculations.

### 2.10. Reusability

To estimate the reusability of the biocatalytic systems, obtained catalysts were applied for the reaction described in Section 2.7, under 40 °C and PBS pH 7. After the first cycle, the mats were washed with the buffer solution at pH 7 and transferred to new vials, to which the same amounts of fresh reagents were added, and the reaction was carried out under the same conditions as before. A total of 5 consecutive cycles were carried out for each mat. The activity of each tested biocatalyst after the first cycle was assumed as 100% of relative activity, becoming a base for other cycles.

### 2.11. Enzymatic Resolution of Racemic Citalopram

Since citalopram’s highest psychotropic effect on the human body appears in the S-form, we conducted an enzymatic, greener resolution of the racemic citalopram. The reaction was carried out in a membrane reactor (Amicon 8010, Amicon, Warsaw, Poland), with the use of the produced HIGH PVC MOF-lip-CA system as a catalyst (r = 2.5 cm). In the recirculated batch reactor with stirring, 5 mL of 40 mM racemic citalopram dissolved in acetonitrile and 1.66 mL of vinyl acetate as the acetyl donor were placed. The flow rate was established and sustained at 1 mL/min by a peristaltic pump. Progress of the reaction was monitored after 1, 8, 12, and 24 h by collecting the samples for HPLC analysis.

## 3. Results and Discussion

### 3.1. Physicochemical Characterization

**Figure 1:**
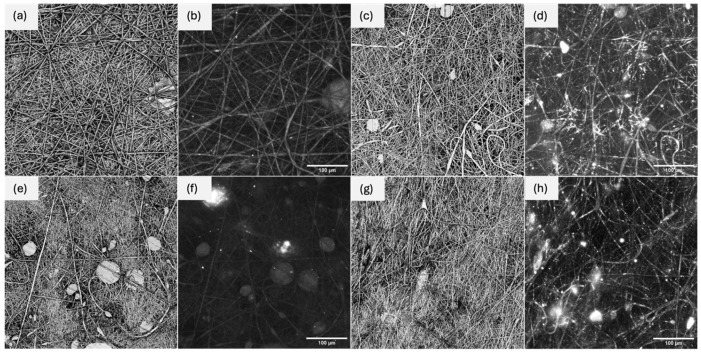
CLSM photos of the electrospun mats fabricated with low-molecular-weight PVC (LOW PVC): (**a**,**b**) pristine and (**c**,**d**) with the addition of MOF, and with high-molecular-weight PVC (HIGH PVC): (**e**,**f**) pristine and (**g**,**h**) with the addition of MOF. Photos were taken in material mode (**a**,**c**,**e**,**g**) and fluorescent mode (**b**,**d**,**f**,**h**).

The aim of using a confocal microscope was to determine the morphological structure of the electrospun mat fibers. It also allowed for the assessment of the potential incorporation of MOF particles into the fiber structure during the electrospinning process of LOW and HIGH PVC mats. In addition, the photos show the quality of the produced fibers, the presence of possible imperfections (polymer drops in the structure), and confirm the formation of a porous structure of the obtained mats that was crucial for use of the electrospun mats as carriers in immobilization. Based on the presented CLSM photos (Figure 1), it can be concluded that high-quality fibers were created during electrospinning. They are dense, creating a network, and especially in the photos of pristine HIGH PVC material, the lack of imperfections in the form of drops is visible.

Due to the fact that polymer molecular weight significantly affects electrospun fiber morphology, low-molecular-weight polymer can lead to formation of imperfections of electrospun fibers (Figure 1a), while higher molecular weight usually results in uniform, smooth fibers due to the increased chain entanglement and solution viscosity [16]. The photos of LOW PVC (Figure 1e) contain spherical areas (imperfections) that might result from non-ideal electrospinning conditions, which caused formation of clusters. The above photos also indicate the incorporation of MOF in the structure of the electrospun mats. This conclusion can also be drawn by comparing photos taken in fluorescence mode for mats spun from LOW PVC without and with MOF (Figure 1f,h) and from HIGH PVC without and with MOF (Figure 1b,d). In the case of mats containing the UiO-66-NH_2_ MOF additive, bright shapes can be seen in fluorescence mode, which are not present in the case of mats spun without additives. In addition, photos in fluorescence mode of mats from HIGH PVC contain more bright inclusions (MOF particles) than mats from LOW PVC. This indicates more effective incorporation of MOF into the structure of a higher-molecular-weight polymer. This is probably the result of the increase in polymer adhesiveness with increasing molecular weight. What is worth mentioning is that even though the MOF is successfully incorporated in the fiber’s structure, it does not change its morphology. The fibers stay uniform and well spun. This is a surprising observation, as previously it was reported by Hasheem et al. that pristine PVC fibers are smooth and continuous, while MOF-loaded fibers display embedded MOF aggregates [17]. However, they noticed that the presence of MOF aggregates is directly related to the specific MOF concentration, because at higher MOF loadings (20%), bead-like structures appear, indicating instability in the electrospinning jet and imperfections in the fiber network, due to the change of conductivity and viscosity of the polymer solution. They concluded that MOFs are evenly distributed at lower loadings but aggregate at higher concentrations.

**Figure 2:**
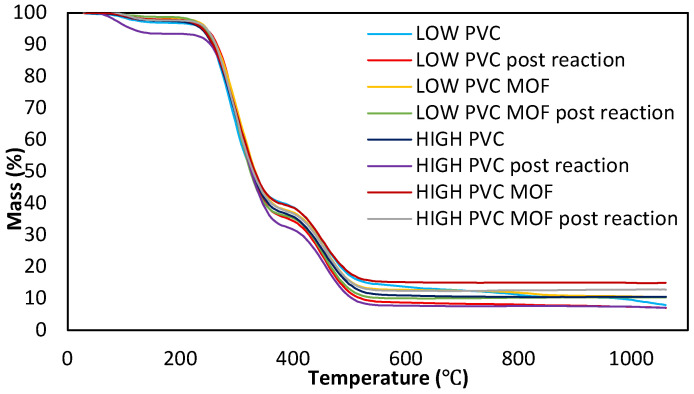
Effect of additive loading (MOF) on the thermal stability of electrospun PVC mats.

Based on the CLSM images presented in this study, it might be assumed that the performed electrospinning process allowed formation of the desired porous structure composed of nanofibers, which provided a large surface area for enzyme immobilization. Besides the structure, also the thermal stability of the material plays a key role during enzyme immobilization and further application, as it can significantly determine the enzyme’s survivability under unfavorable conditions, ensuring protection of the protein structure. Thermogravimetric analysis (Figure 2) allowed us to determine which electrospun mat is the most durable and to check whether the incorporation of MOF into electrospun fibers is permanent and whether contact with an aqueous buffer solution will contribute to its leaching.

It can be stated that thermally, the most stable material turned out to be the electrospun mat made of HIGH PVC with the addition of UiO-66-NH_2_ (Figure 2, maroon line). Further, the influence of MOF on the thermal stability of the material was more visible in the case of this PVC than for LOW PVC. This suggests again that the incorporation of MOF into the organic carrier was more effective for HIGH PVC, so with the increase of the molecular weight of this polymer, its adhesion capacity increased [18]. Moreover, the mat made of HIGH PVC without the addition of MOF showed a slightly smaller weight loss than in the case of the polymer with a lower molecular weight. Hence, it can be assumed that the thermal stability of the carrier is also influenced by the molecular weight of the polymer, because with its increase, the thermal resistance of the fibers increases [19]. Consequently, MOF addition increases not only the thermal stability but also the stability of the material in aqueous solutions, in agreement with previously published studies [17].

### 3.2. Immobilization Characterization

To determine how the addition and combination of the UiO-66-NH_2_ MOF, choline chloride (CC), and choline acetate (CA) ILs, as well as the time of the process, affect the efficiency of the lipase immobilization process, its efficiency was examined (Table 1). The obtained results showed that the highest immobilization efficiency, reaching 52%, was obtained in the case of *Candida* sp. lipase immobilization on a HIGH PVC electrospun material with the addition of MOF in the structure and choline acetate as a substance supporting enzyme immobilization.

The incorporation of MOF in the structure of electrospun mats increased the enzyme adsorption due to the UiO-66-NH_2_ features, such as large surface area and the presence of functional groups that increase the possibility of efficient enzyme attachment. Similar observations were made by Molco et al. after the fabrication of electrospun polymer fibers with in situ grown MOFs (HKUST-1 and ZIF-8) that possessed different functional groups. The MOF crystals grown toward the fiber surface created exposed active sites that significantly enhanced enzyme immobilization and catalytic performance, compared to fibers without MOFs [20]. In our study, it was observed that the addition of ionic liquids affected the immobilization efficiency, which resulted from the tendency of ionic liquids to stabilize the enzymatic structure [21]. The ionic liquid also increased the enzyme affinity to the mat, creating a favorable microenvironment, which ultimately increased the immobilization efficiency. It can also be seen that choline acetate increased the immobilization efficiency more than the addition of choline chloride, which shows that lipase immobilization is dependent on the pH of the solution (choline chloride is more acidic than choline acetate). The pH also affected enzyme conformation and charge distribution, which in turn modulated the enzyme’s affinity for the support surface [22].

Further, the results from Table 2 show that a longer immobilization time also had a positive effect on the activity of biocatalytic systems, because the activity of each tested system was higher in the case of 24 h immobilization than in the case of 1 h immobilization. This is due to the fact that the immobilization efficiency also increased with time and, therefore, with the increase in the number of enzyme particles immobilized on the support, its biocatalytic activity increased [23].

What is worth mentioning is that the immobilization efficiency ranging from 40 to 60% is sufficient, determining a suitable environment for the immobilized enzyme, without the threat of the enzyme overloading, which could cause a significant decrease in the catalytic performance. Data also show that the addition of choline acetate increased the activity of the systems more than the addition of choline chloride, which is not only associated with a higher immobilization efficiency in the systems to which choline acetate was added but also with a suitable pH provided by choline acetate in which lipase reached the highest relative activity. Lipase showed higher activity at alkaline pH, while choline chloride was more acidic than choline acetate, leading to lower lipase activity [24]. The highest activity was obtained using a HIGH PVC mat with the addition of MOF and after adding choline acetate in the immobilization process. Consequently, the use of the UiO-66-NH_2_ additive during the formation of electrospun mats as well as the addition of this IL in the immobilization process had a positive effect on the enzymatic activity of the immobilized lipase.

**Figure 3:**
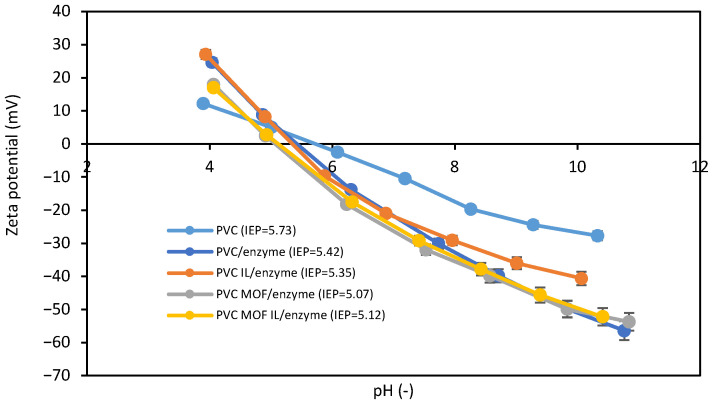
Zeta potential over varying pH levels of the fabricated biocatalysts (based on HIGH PVC) with and without the choline acetate. The data are presented as a mean value from three experiments, and error bars represent the standard deviation.

Zeta potential was evaluated in terms of enzyme immobilization and the influence of ILs on the process (Figure 3). At a pH between 5 and 10, the electrokinetic potential of all fabricated membranes showed the same course with the zeta potential of the membranes with an immobilized enzyme with negative values.

Moreover, all systems were characterized by an isoelectric point (IEP) at a similar pH between 5 and 6. However, the zeta potential of the HIGH PVC membrane ranged from 0 to ± 30 mV, which indicates the electrokinetic instability of this system [25]. On the other hand, the systems with an immobilized enzyme were characterized by good electrokinetic stability (zeta potential below −30 mV) at alkaline pH. A slight deterioration in the stability of the HIGH PVC-lip-CA system, compared to the HIGH PVC-lip system, could also be observed. This behavior can be explained, for example, by the higher accessibility of the unmodified fiber for the aqueous measurement solution. It should also be noted that the HIGH PVC-MOF-lip and HIGH PVC-MOF-lip-CA systems were characterized by very similar zeta potential values in the entire pH range studied, suggesting that both systems had similar accessibility of the aqueous measurement solution. It is also worth noting that the MOF-modified systems were characterized by electrokinetic stability in the widest pH range from 7.5 to about 10. A similar result was observed by Wang et al., who showed that the addition of MOF (UiO-66) improved the stability of ultrafiltration membranes [26]. Moreover, similarly to the presented studies, they observed a decrease in the zeta potential value and an improvement in the stability of the modified membranes with increasing pH. The assumption that incorporation of an appropriately selected modifier to polymer membrane improves its stability was also confirmed by studies conducted by EL Hady et al., in which the membrane modified with CoFe_2_O_4_@SiO_2_ core-shell nanoparticles was characterized by better stability (zeta potential below –30 mV) than the pure PVC membrane (zeta potential between 0 and –30 mV) [27]. Thus, based on the presented experimental and literature data, it can be stated that modification with an appropriately selected compound, for example, a metal–organic framework (MOF), affects the value of the zeta potential and improves the electrokinetic stability of the systems.

The results obtained in the above study and data on immobilization efficiency led to the rejection of further studies on the effect of choline chloride addition on the process. Both the efficiency of the immobilization and the activity of the systems increased more with the use of choline acetate. Moreover, it turned out that a longer process time (24 h) increased its efficiency, and further studies were carried out on biocatalysts obtained as a result of 24-h immobilization. The systems analyzed in subsequent studies were based on a biocatalyst with immobilized lipase with and without the addition of choline acetate (25 µL), for comparison.

### 3.3. Biocatalysts’ Evaluation

Free enzymes are often sensitive to high temperatures, at which they denature. The aim of the immobilization process was to increase their thermal stability so that they can be used in industrial processes often carried out at elevated temperatures. In the study on comparison of free and immobilized lipase, the concentration of the free enzyme was maintained equal to that of the immobilized enzyme to ensure a reliable comparison of their catalytic performance under identical conditions.

**Figure 4:**
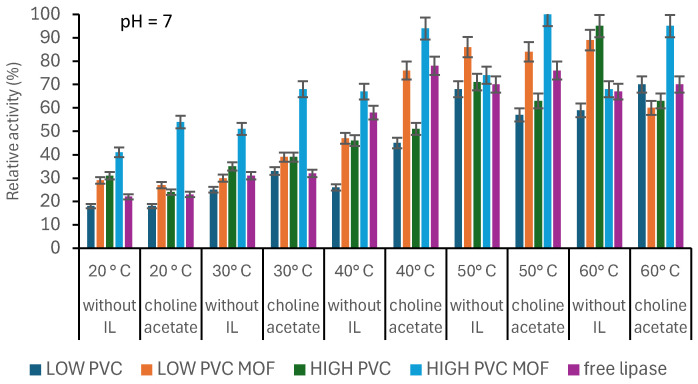
The effect of the temperature on the relative activity of produced biocatalysts and free lipase. The data are presented as a mean value from three experiments, and error bars represent the standard deviation.

**Figure 5:**
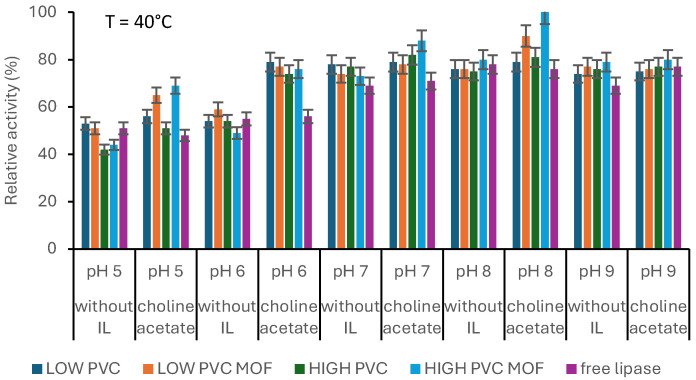
The effect of the pH on the relative activity of produced biocatalysts (with and without ionic liquid (IL)) and free lipase. The data are presented as a mean value from three experiments, and error bars represent the standard deviation.

The most active biocatalyst turned out to be the system consisting of an enzyme immobilized on an electrospun PVC mat of high molecular weight with MOF added at 50 °C (Figure 4), but generally it was observed that lipase showed higher activity at higher temperatures (50 °C and 60 °C) than at lower temperatures (20 °C, 30 °C, and 40 °C). Figure 5 also shows that immobilized lipase showed increased thermal tolerance, resulting in the possibility of using it in processes occurring at higher temperatures. Higher temperatures increased the movement of molecules moving in solution, which increased the affinity of the enzyme for the substrate and, therefore, the reaction was more efficient [28].

Moreover, materials containing MOF formed during immobilization with choline acetate showed higher activity than free lipase at almost all temperatures. This indicates that the hydrophobicity of IL creates a suitable microenvironment around the active site of lipase, thereby increasing its catalytic activity [29]. As stated by Waggett et al., consciously picked ionic liquids can help to maintain low water activity, which is crucial for lipase activity in, e.g., non-aqueous systems. Lipases typically require a thin layer of water for activity, but excess water can lead to hydrolysis of the desired product or enzyme denaturation [30]. The interfacial activation mechanism, where the “lid” domain of lipases undergoes conformational changes for activity, can be boosted with hydrophobic ILs, which can stabilize the open conformation of the lipase lid, exposing the active site and promoting substrate binding and increased relative activity [31,32]. As stated before, the pH of the solution had a significant impact on the activity of the biocatalytic systems (Figure 5). The most active biocatalyst was noticed at pH 8 and turned out to be the HIGH MOF system.

**Figure 6:**
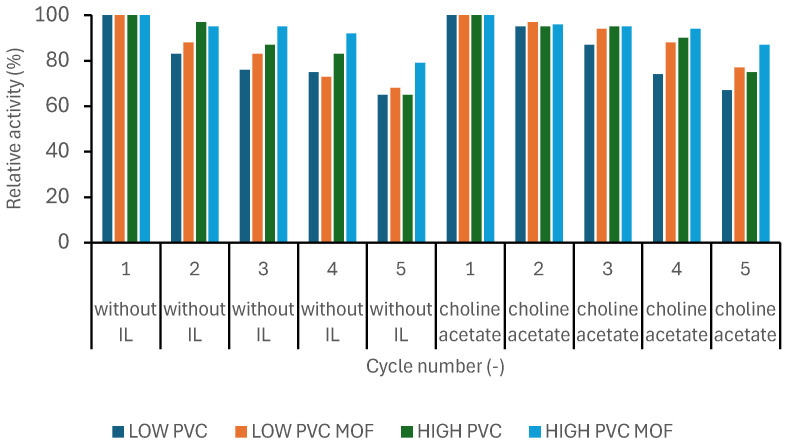
The dependence of the relative activity of the produced biocatalysts (with and without ionic liquid (IL)) during the reusability performance. The data are presented as a mean value from three experiments, and error bars represent the standard deviation.

As described previously, immobilization enables simple separation of the biocatalyst from the post-reaction mixture and, therefore, facilitates multiple uses. The conducted study allowed us to determine not only the possibility of reuse but also whether the enzyme’s action in subsequent cycles of use was effective. These features are especially important at the time of potential use of biocatalytic systems on an industrial scale. The decrease in biocatalyst activity with each cycle of use was greater for systems without ionic liquid than for systems with choline acetate (Figure 6). This shows that adding ionic liquid during the immobilization process increased the stability of the obtained systems by strengthening the enzyme binding to the support [33]. This may also result in lower enzyme leaching during each reaction in solution. Also, the activity of LOW PVC mats was lower compared to the HIGH PVC mats in systems without ionic liquid and MOF addition, which shows that the possibility of multiple uses is also influenced by the choice of the polymer itself, due to the morphological and stability features of the produced support material [34]. The effect of the UiO-66-NH_2_ addition on the reusability was also visible—the activity of systems with MOF addition had a smaller decrease in activity with each cycle of use. This dependency might result from higher amounts of immobilized enzyme due to the higher surface area and groups allowing a proper attachment of protein to the fiber [35]. The smallest decrease in activity and, consequently, the highest reusability was demonstrated by the biocatalyst consisting of an enzyme immobilized on HIGH PVC with the addition of MOF and choline acetate.

At slightly acidic pH, the addition of choline acetate increased the activity of the immobilized enzyme more than the free enzyme. Consequently, the addition of this IL did not increase the resistance of the free enzyme to low pH, where the protective effect of the support material provided such resistance [36]. This may indicate that IL increases the stability and activity of biocatalytic systems. The addition of choline acetate increased the activity of biocatalytic systems in the entire pH range studied, which shows that the immobilization of *Candida* sp. lipase with this IL is able to expand the range of its possible applications. The type of immobilization applied in this study can be classified as IL-assisted physical adsorption (physisorption/electrostatic adsorption). The mechanism is based on non-covalent interactions between the enzyme, the ionic liquid, and the MOF/PVC hybrid surface. The ionic liquid (IL) acts as a stabilizing and activating agent, enhancing enzyme orientation and catalytic performance due to the interfacial activation phenomenon that occurs in hydrophobic conditions, while also protecting the enzyme from denaturation. Such IL-assisted physical immobilization on hybrid supports contributes to maintaining the structural integrity of the enzyme and improving its thermal and operational stability. This may further explain the high activity and durability of the biocatalytic systems observed under various pH conditions [37]. Finally, the immobilized systems were also less active at acidic pH (5) than at alkaline pH (8 and 9), which is characteristic for free lipase and confirms that no significant changes in the enzyme structures occurred during immobilization.

**Figure 7:**
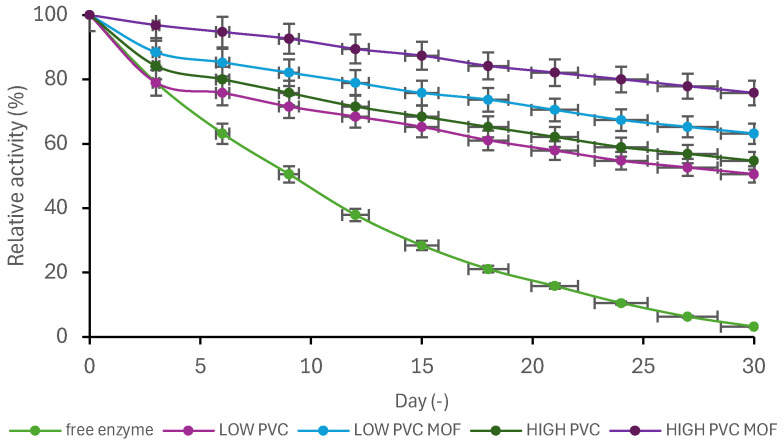
Storage stability of the free lipase and produced biocatalytic systems. The data are presented as a mean value from three experiments, and error bars represent the standard deviation.

Storage stability tests are essential to ensure the long-term integrity of the produced biocatalytic systems. The fabricated systems were stored in a buffer solution at pH 7, which meant that the storage environment was constantly interacting with them. This interaction could cause the enzyme to wash out from the carrier material, which could have a direct impact on the activity of the obtained systems. Based on the obtained results (Figure 7), the system that lost its activity the slowest, and therefore was the most stable in the test, was the one in which the carrier was high-molecular-weight PVC with the addition of MOF. UiO-66-NH_2_ as a metal–organic framework has a large surface area, which allowed for immobilization of a larger amount of enzyme on mats containing it. In addition to increasing the immobilization efficiency, it can also be stated that MOF hindered the enzyme from being washed out of mats stored in aqueous solutions. However, not only the addition of UiO-66-NH_2_ had an impact on the stability of mats with immobilized enzyme, but also the molecular weight of the polymer from which they were electrospun. Biocatalysts made of HIGH PVC showed greater stability than those made of low-molecular-weight PVC, which shows that electrospun fibers made of HIGH PVC have greater strength and a greater affinity to the enzyme. It is also directly related to the morphology of the electrospun fibers, since the HIGH PVC ones were characterized with a smoother and more uniform structure, compared to LOW PVC. For this reason, the enzyme may leak out more slowly from these mats and, therefore, their catalytic activity will also decrease more slowly. The free CALB rapidly lost its catalytic activity (remaining activity ~3%) mainly due to conformational destabilization in aqueous solution. Storage in 50 mM sodium phosphate buffer (pH 7.0, Na_2_HPO_4_/NaH_2_PO_4_) without any stabilizing additives, such as polyols or proteins, likely promoted gradual unfolding and aggregation. Moreover, phosphate ions can interact with charged amino acid residues on the enzyme surface, subtly altering its electrostatic balance and hydration shell, which may further contribute to conformational drift and loss of the active form. The obtained results also show the advantages of the immobilization process itself. The enzyme immobilized on the support had greater stability in each type of electrospun mat than free lipase. The immobilization process helped in saving a higher catalytical activity of the enzyme, due to the protective environment, which is not seen with the native form of the enzyme, where the molecule is directly exposed to the process environment. In another study in which lipase was encapsulated in a ZIF-8 structure, it was shown that after 25 days of incubation in phosphate buffer, it retained as much as 90% of its initial activity [38]. For comparison, in that study, free lipase and sonicated lipase retained only 68% and 66% of activity, respectively. Such high stability was attributed to the protective effect of MOF, which isolated the enzyme from the unfavorable effects of the chemical environment and minimized deformations within the active site of the enzyme. Presented results suggest that the immobilization method can significantly increase the stability of enzymes but can also affect their activity and active center availability for the possible substrate. It is worth emphasizing that after 30 days of storage stability, the system immobilized on HIGH MOF with the addition of choline acetate IL maintained 80% of activity compared to 3% of the free enzyme, demonstrating significantly increased enzyme protection.

### 3.4. Enzymatic Resolution of Racemic Citalopram

**Figure 8:**
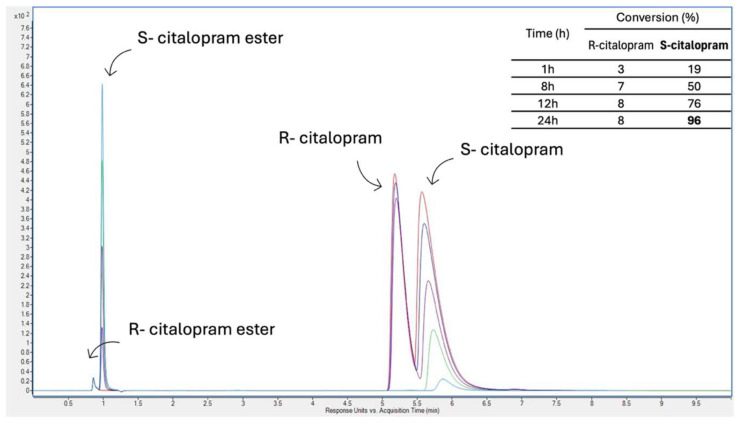
Chromatogram generated after HPLC analysis of rac-citalopram resolution and the product S-citalopram ester. Colors on the chromatogram correspond to specific samples collected after certain times (from the top): red—0 h, navy blue—1 h, purple—8 h, green—12 h, and light blue—24 h.

After the characterization, one biocatalytic system was chosen for further application. HIGH PVC MOF-lip-CA was applied for tests in the recirculated batch membrane reactor to estimate its catalytic activity in the resolution of racemic citalopram to obtain the enantiomerically pure product S-citalopram ester (see Figure 8).

**Figure 9:**
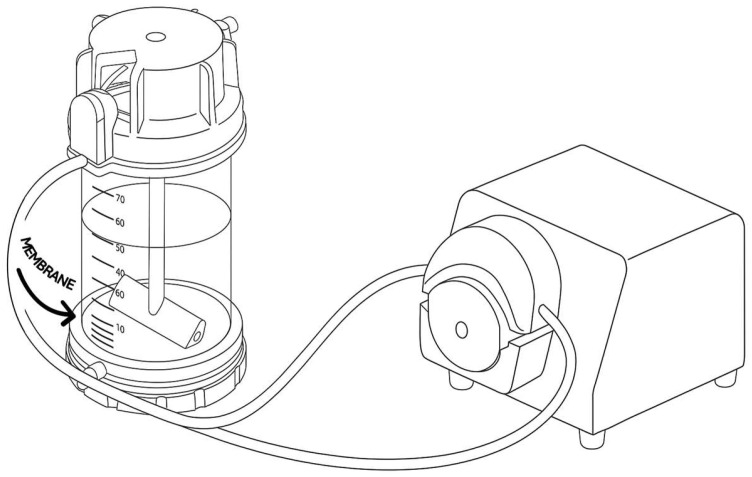
Scheme of the membrane bioreactor for the asymmetric resolution of racemic citalopram.

Enzymatic resolution of racemic citalopram in a membrane reactor yielded the desired product, citalopram S-ester (see scheme of the reactor on Figure 9). As can be seen in the chromatogram (Figure 8, right-hand peak), the concentration of S-citalopram decreased, while the concentration of S-ester increased, indicating successful conversion of the substrate to the product. An even more important observation was the slight loss of R-citalopram and minimal amount of R-ester in the final sample, demonstrating the high enantioselectivity and stereoselectivity of the biocatalytic system applied in the membrane reactor. Here, 1 h after starting the reaction, conversion of S-citalopram reached 19%, followed by 50% after 8 h and 76% after 12 h. In addition, enzyme stability in the recirculated batch membrane reactor was evaluated. The immobilized lipase maintained high catalytic performance throughout the process, exhibiting only about 8% loss of its initial activity after the reaction was completed. This result confirms that the enzyme remained stable under reaction conditions, which often impose mechanical and diffusional stresses that can lead to deactivation. The observed stability indicates strong enzyme–support interactions and an effective immobilization strategy that preserves enzyme conformation and activity over prolonged operation. Such robustness is a crucial factor for potential industrial applications, as it ensures reproducibility, cost efficiency, and long-term operational stability of the biocatalytic system.

As a result, an enantiomeric excess of S-ester over R-ester of >93% was obtained, with 95% conversion of S-citalopram after 24 h of reaction. Over the tested conversion time, the conversion of R-citalopram did not exceed 8%, proving the validity of the biocatalytic system used and the appropriate selection of the enzyme strain for the reaction. The obtained results show a promising application possibility and a great upscaling potential. Similar observations were made by Wang et al., where racemic (±)-1-phenylethanol, a citalopram intermediate, was resolved using *Candida antarctica* lipase B immobilized on silanized silica in an organic solvent with vinyl acetate. The process achieved around 50% conversion, >99% enantiomeric excess of (R)-1-phenylethanol, with high enantioselectivity (E > 200) [39].

## 4. Conclusions

This study successfully developed a highly efficient and stable biocatalytic system by integrating electrospun high-molecular-weight PVC mats with UiO-66-NH_2_ and choline acetate IL. The MOF incorporation significantly enhanced the surface area and functional group density of the support, enabling high enzyme loading and minimized leaching, while the IL created a stabilizing microenvironment that broadened the lipase’s operational range across pH and temperature. Optimal immobilization (24 h) yielded an exceptional 52% efficiency and 100% activity recovery, with the biocatalyst demonstrating peak performance under alkaline conditions (pH = 8–9) and elevated temperatures (50–60 °C). Notably, the system exhibited superior storage stability and reusability, critical for industrial applications. A key achievement was the system’s application in a membrane reactor for the enantioselective resolution of racemic citalopram, achieving 93% enantiomeric excess at 95% conversion of S-citalopram, a milestone for sustainable pharmaceutical synthesis. The polymer’s molecular weight was identified as a critical factor, with higher weights improving the fiber durability and immobilization efficiency. By combining the MOF-enhanced carrier design, IL-mediated enzyme stabilization, and membrane reactor technology, this work advances green biocatalysis, offering a scalable, eco-friendly alternative to traditional chemical processes in drug manufacturing.

## Figures and Tables

**Table 1:** Immobilization efficiency influenced by time and addition of different ILs.

Immobilization Efficiency (%)			
	Without IL	Choline Chloride	Choline Acetate
**1 h**			
**LOW**	23 ± 0.9	29 ± 0.5	34 ± 1.1
**LOW MOF**	24 ± 1	26 ± 0.9	37 ± 0.8
**HIGH**	21 ± 0.8	19 ± 0.1	40 ± 0.8
**HIGH MOF**	25 ± 0.05	27 ± 0.1	38 ± 0.2
**24 h**			
**LOW**	22 ± 0.8	21 ± 1	42 ± 1.2
**LOW MOF**	28 ± 0.2	33 ± 0.9	35 ± 0.6
**HIGH**	27 ± 0.3	45 ± 0.7	48 ± 0.2
**HIGH MOF**	47 ± 0.05	45 ± 0.2	52 ± 0.1

**Table 2:** Comparison of the effect of immobilization time, type of support, and type of ILs added on the catalytic activity of the produced systems.

Activity Recovery (%)			
	Without IL	Choline Chloride	Choline Acetate
**1 h**			
**LOW**	30 ± 1.1	37 ± 1	40 ± 0.9
**LOW MOF**	34 ± 0.6	38 ± 1.1	44 ± 0.8
**HIGH**	32 ± 1.1	44 ± 0.5	52 ± 1
**HIGH MOF**	59 ± 0.2	60 ± 0.9	74 ± 0.1
**24 h**			
**LOW**	37 ± 0.4	39 ± 0.8	49 ± 1
**LOW MOF**	45 ± 0.2	44 ± 0.4	57 ± 1
**HIGH**	51 ± 0.2	49 ± 0.5	58 ± 0.3
**HIGH MOF**	76 ± 0.2	77 ± 0.4	100 ± 0.1

## Data Availability

The data that supports the findings of this study is available from the corresponding authors upon request.

## Abbreviations

The following abbreviations are used in this manuscript:

API	Active pharmaceutical ingredient
CA	Choline acetate
CC	Choline chloride
DCM	Dichloromethane
DMF	N,N-dimethylformamide
IL	Ionic liquid
MOF	Metal–organic framework
pNPP	Paranitrophenyl palmitate
PVC	Poly(vinyl chloride)
